# Galectin-3 as a Potential Prognostic Biomarker for COVID-19 Disease: A Case-Control Study

**DOI:** 10.7759/cureus.28805

**Published:** 2022-09-05

**Authors:** Emre Karsli, Damla Anabarli Metin, Omer Canacik, Ramazan Sabirli, Buse Kaymaz, Ozgur Kurt, Aylin Koseler

**Affiliations:** 1 Emergency Medicine, Faculty of Medicine, İzmir Tınaztepe University, Izmir, TUR; 2 Emergency Medicine, Faculty of Medicine, Karabuk University, Karabuk, TUR; 3 Emergency Medicine, Sisli Memorial Hospital, Istanbul, TUR; 4 Emergency, Faculty of Medicine, Izmir Bakircay University, Izmir, TUR; 5 Medical Biotechnology, Acibadem Mehmet Ali Aydinlar University School of Medicine, Institute of Health Sciences, Istanbul, TUR; 6 Microbiology, Acibadem Mehmet Ali Aydinlar University School of Medicine, Istanbul, TUR; 7 Biophysics, Pamukkale University, Denizli, TUR

**Keywords:** inflamation, clinical infectious disease, covid-19 pneumonia, covid-19, galectin-3

## Abstract

Background

Recent studies have investigated the importance of Galetin-3 in inflammation, fibrosis, cell proliferation, cardiac disease, diabetes, and tumor formation.

Aims

This study aims to investigate the role of the Galectin-3 level in the diagnosis of COVID-19 pneumonia and the value of the Galectin-3 level in predicting the clinical course of the patient.

Methods

This study employed a prospective, case-control study design and was conducted at Bakircay University Ciğli Training and Research Hospital. A total of 100 patients (40 had moderate and 60 had severe/critical COVID-19 disease according to World Health Organisation guidelines) and 50 non-symptomatic healthy volunteers participated in the study. Blood samples were taken from patients at the time of hospital admission, after which serum was isolated. Following the isolation of serum, Galectin-3 levels were evaluated using the enzyme-linked immunosorbent assay (ELISA) method.

Results

The serum Galectin-3 level was measured as 13.57 (10.9-16.4) ng/mL in the control group, 13.52 (10.69-16.6) ng/mL in the moderate disease group, and 11.65 (6.09-14.33) ng/mL in the severe/critical disease group. Serum Galectin-3 levels were significantly lower in the severe/critical disease group compared to the control and moderate disease groups (p=0.001 and p=0.019, respectively).

Using ROC analysis, a larger area under the curve (AUC) for the serum Galectin-3 levels of the control group (AUC=0.622, 95% CI =0.529-0.714; p=0.015) was calculated compared to the COVID-19 patient group for the diagnosis of COVID-19 disease. The Galectin-3 level was found to be 75% sensitive and 50% specific at a cut-off level of 11.3 ng/mL in predicting the need for ICU treatment.

Conclusion

Galectin-3 levels may be a beneficial biomarker in predicting the clinical severity of COVID-19 disease when used in conjunction with other known biomarkers, at the time of admission to the emergency department (ED).

## Introduction

The COVID-19 infection, which is transmitted from person to person through droplets and can lead to serious clinical conditions such as pneumonia, acute respiratory distress syndrome (ARDS), sepsis, and septic shock from the clinic of upper respiratory tract infection, continues to pose a significant problem worldwide [1,2].

In the follow-up of inflammatory processes caused by COVID-19, both clinical parameters and many laboratory parameters are evaluated. These parameters are used for estimating high-risk patients, determining the response to treatment, determining the intensive care unit (ICU) admission criteria, outcome prediction, and determining the discharge criteria [1].

In addition to known inflammatory markers such as C-reactive protein (CRP), procalcitonin, interleukins, neutrophil-to-lymphocyte ratio, serum amyloid protein, erythrocyte sedimentation rate, and ferritin, previous studies published during the pandemic have examined markers that may be associated with inflammation such as Annexin A1, S100B, and calprotectin for diagnosis and prognosis [2-5].

Galectin-3 is a chimera-type galectin of approximately 30 kDa and is expressed in the nucleus, cytoplasm, mitochondria, cell surface, and extracellular region in the cell. Galectin-3 and other galectin proteins are used in cell-cell communication and cell-matrix communication, and it plays a role in cell growth, cell differentiation, angiogenesis, and apoptosis. Recent studies have investigated its importance in inflammation, fibrosis, cell proliferation, cardiac disease, diabetes, and tumor formation [6-11]. A study conducted with mice found that stimulation of Galectin-3 activity was effective on direct bacteriostatic activity in severe pneumococcal infection and provided better clinical results [12].

Although a prospective study has been conducted to evaluate the role of Galectin-3 inhibitors in the treatment of COVID-19 previously, to the authors’ knowledge, there is no study examining the change of Galectin-3 protein level at the clinical level in COVID-19, and few studies have examined the usability of Galectin-3 level in the diagnosis of COVID-19 pneumonia [13,14]. This study aims to investigate the role of the Galectin-3 level in the diagnosis of COVID-19 pneumonia and the value of the Galectin-3 level in predicting the clinical course of the patient.

## Materials and methods

Study design and study population

The present study is a prospective case-control study, and the ethics approval was obtained from the Ethics Committee of Pamukkale University (Numbered: E-60116787-020-67194). The study was conducted at Bakircay University Cigli Training and Educational Hospital. All procedures carried out on patients were in compliance with the Helsinki Declaration.

The patient groups and the healthy control group were informed in detail about the study, and they were requested to complete the written consent forms before participating in the study. Study groups were determined according to the inclusion and exclusion criteria. Patients who were clinically confirmed as COVID-19 infection according to World Health Organization (WHO) guidelines using a positive reverse transcriptase polymerase chain reaction (RT-PCR) test were included in the study. Participants were grouped in the moderate COVID-19 disease group (N=40), the severe/critical COVID-19 disease group (N=60), and the healthy control group (N=50). according to WHO guidelines and those who gave their written consent were included in the study [15]. The healthy control group included healthy volunteers with no history or diagnosis of any acute or chronic disease and infection and no known drug use.

Inclusion criteria

Patient Groups

Patients whose diagnoses of COVID-19 infection were confirmed by positive RT-PCR in ED [15].

Control Group

Subjects with no history of a known disease, no infectious symptoms, no drug use, and who provided written consent were included in the study.

Exclusion criteria

Patients who were diagnosed with heart, kidney or liver failure, who had a history of acute pulmonary embolism, deep venous thrombosis or chronic inflammatory disease, and who were pregnant were excluded from the study. 

Clinical evaluation

**Figure 1:**
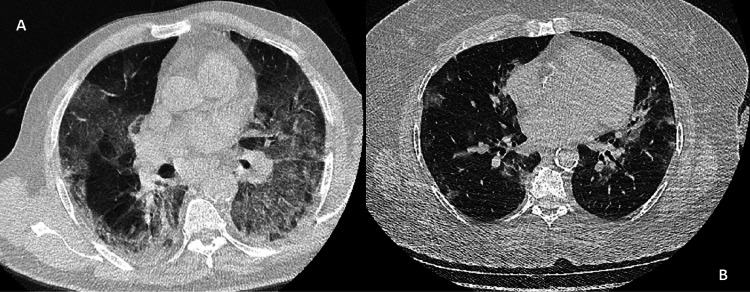
Radiological examples of COVID-19 patients (A) Severe COVID-19 pneumonia. (B) Moderate disease.

The subjects included in the present study were clinically evaluated using WHO diagnosis and treatment guidelines for COVID-19. The patient management algorithms were administered due to the updates of these guidelines. The patients were categorized as moderate disease and severe/critical disease groups according to WHO guidelines. The severe/critical disease group includes the patients who have severe pneumonia or ARDS signs (adolescent or adult with clinical signs of pneumonia [fever, cough, dyspnoea, fast breathing] plus one of the following: respiratory rate > 30 breaths/min; severe respiratory distress; or SpO_2_ < 90% on room air and oxygenation impairment findings) [15]. Radiological examples were given in Figures 1A, 1B.

To evaluate the clinical severity, CURB-65 scores of patients were calculated as indicated in the literature. The CURB-65 score is a scoring system used in the evaluation of pneumonia [16].

 Healthy group (control group)

This group included subjects who gave their written consent to participate in the study, who had no infection history within the last two weeks, no history of any particular medication, no history or diagnosis of any disease, and who were admitted to the emergency department (ED) with complaints other than infectious issues.

Data collection

Demographic information and vital findings of the subjects, and their laboratory findings (hemogram, C-reactive protein (CRP), liver function tests (aspartate aminotransferase, alanine aminotransferase), creatinine, blood urea nitrogen (BUN), D-dimer, creatinine kinase-MB, highly sensitive troponin T (hsTnT), blood gas analysis parameters, and hospitalization location ICU or not were recorded in the data set.

Galectin-3 level measurement

Venous blood samples that were taken when the patients were admitted to ED were withdrawn into a dry test tube. Samples were centrifugated for 10 minutes at 4,000 rpm. Serum samples obtained from centrifugation were collected for laboratory analysis. Serum Galectin-3 levels were analysed using a commercially available Galectin-3 enzyme-linked immunosorbent assay (ELISA) Kit (Human Galectin-3 ELISA Kit, Elabscience, Catalog No: E-EL-H1470), per the manufacturer’s protocol.

Statistical analysis

Given that a similarly organized reference study did not exist, a power analysis was performed in line with the presumptions. The results revealed that at least 88 people (min. 44 for each cohort) were needed to achieve 95% power at a 90% confidence interval, assuming that the projected effect size would be high (f=0.7). The dataset was analysed by using the SPSS package programme.

A Kolmogorov-Smirnov test was conducted to calculate the distribution type of the continuous variables. The continuous variables were presented as mean ± standard deviation or median (IQR). Kruskal-Wallis or Mann-Whitney U tests were used for analysing independent and non-parametric variables. Receiver operating characteristic (ROC) curve analysis was used for the discriminant performance serum Galectin-3 levels. The significance level was defined as p < 0.05 for all analyses.

## Results

Symptom duration time was statistically higher in the severe/critical disease group than in the moderate disease group (7.5±1.7 and 5.9±1.1 days, respectively) (p=0.02). The post-hoc power analysis showed that the effect size of the Galectin-3 concentrations for the differences between the two groups (patients and control) was moderate-high (f=0.63), and the power level observed for this effect size was 95%, and the reliability level was 95.2%.

Serum Galectin-3 levels were measured as 13.57 (10.9-16.4) ng/mL in the control group; 13.52 (10.69-16.6) ng/mL in the moderate disease group; and 11.65 (6.09-14.33) ng/mL in the severe/critical disease group. Serum Galectin-3 levels were significantly lower in the severe/critical disease group compared to the control and moderate disease groups (p=0.001 and p=0.019, respectively) (Table 1).

**Table 1:** Galectin-3 levels of the study groups *p-value derived from Kruskal-Wallis' test and refers to the comparison between whole the groups **p-value is derived from the Mann-Whitney U test and refers to the comparison between control and moderate disease groups. ***p-value is derived from the Mann-Whitney U test and refers to the comparison between control and severe/critical disease groups. ****p-value is derived from the Mann-Whitney U test and refers to the comparison between moderate disease groups and severe/critical disease groups.

	Controls (N=50)		Moderate Disease Group (N=40)			Severe/Critical Disease Group (N=60)		P-value
	Mean±SD	Median (IQR)	Mean±SD		Median (IQR)	Mean±SD	Median (IQR)	
Galectin-3	15.78±10.8	13.57 (10.9-16.4)	12.58±5.34	13.52 (10.69-16.6)		9.95±5.15	11.65 (6.09-14.33)	^*^p= 0.003
								^**^p= 0.548
								^***^p= 0.001
								^****^p= 0.019

Subjects included in patient groups and healthy control groups were matched by means of age and gender (p=0.686 and p=0.823, respectively) (Table 2).

**Table 2:** Clinical data and comorbidity data of the groups p-values are derived from the Mann-Whitney U test. *p-values are derived from chi-square test **p-values are derived from one-way ANOVA test SBP: Systolic blood pressure; DBP: Diastolic blood pressure.

		Controls (N=50)	Moderate Disease Group (N=40)	Severe/Critical Disease Group (N=60)	P-value
Gender, N(%)	Male	19	18	22	*0.686
	Female	31	22	38	
Comorbidities, N(%)					
Diabetes Mellitus			16 (40%)	26 (43.3%)	*0.766
Hypertension			13 (32.5%)	25 (41.6%)	*0.405
Coronary artery disease			6 (14.2%)	6 (18.7%)	*0.41
		Mean±SD or Median(IQR)			
Age (year)		65.32±16.1	64.05±13.5	65.8±12.67	**0.823
CURB-65 Score			2 (1-2)	3 (2-4)	0.0001
Body temperature (^0^C)			36.75 (36.5-37.1)	36.8 (36.3-37)	0.802
Heart Rate (beat/min)			88 (78.25-95.75)	103 (93-120)	0.003
Respiratory Rate			24 (20-28)	27 (20-38)	0.281
sPO_2_			94 (90-96.75)	85 (78-92)	0.0001
SBP (mm/Hg)			137 (116-156.75)	130 (114-143)	0.106
DBP (mm/Hg)			73.5 (65.25-84.5)	76 (70-82)	0.732

Significant findings and clinical data for the study groups are given in Table 2, and Table 3 presents the laboratory parameters of the subjects.

**Table 3:** Laboratory parameters of the patient groups p-values are derived from the Kruskal-Wallis test *p-values are derived from Student’s t-Test IQR: Interquartile range; WBC: White blood cell; NLR: Neutrophil leukocyte ratio; CRP: C-reactive protein; BUN: Blood urea nitrogen; AST: Aspartate transaminase; ALT: Alanine transaminase; hsTnT: Highly sensitive troponin T; CKMB:  Creatinine kinase MB; pH: Power of hydrogen; pCO_2_: Partial carbon dioxide pressure; HCO_3_: Bicarbonate.

	Moderate Disease Group (N=40)	Severe/Critical Disease Group (N=60)	P-value
	Mean±SD or Median (IQR)	Mean±SD or Median (IQR)	
WBC (K/μL)	5.52 (4.48-6.85)	9.47 (7.76-14.2)	0.0001
Hemoglobin (g/dL)	13.21±1.52	13.55±1.69	^*^p=0.036
Platelete (K/μL)	205.56±57.33	263±94.83	^*^p=0.007
NLR	3.17 (2.1-4.38)	8.27 (3.67-10.2)	0.0001
CRP (mg/L)	31.37 (10.01-92.28)	123 (77.8-196.7)	0.0001
BUN (mg/dL)	33 (25-45)	55 (34.7-77)	0.004
Creatinine (mg/dL)	0.85 (0.79-1.06)	1.23 (0.91-1.52)	0.321
AST (U/L)	27 (21-33)	30 (21.5-46)	0.626
ALT (U/L)	20 (13-29)	26 (20.5-44.5)	0.005
D-Dimer (ng/mL)	530 (290-1120)	1,820 (718-4,400)	0.0001
hsTnT (μg/L)	11.78 (6.7-20.37)	25.5 (8.25-64.67)	0.001
CKMB (ng/mL)	1.39 (0.83-2.45)	2.67 (1.64-7.4)	0.001
pH	7.41 (7.37-7.44)	7.39 (7.3-7.44)	0.437
pCO_2 _(mmHg)	38.9 (35.8-44)	42.2 (35.87-62)	0.045
Lactate (mmol/L)	1.8 (1.2-2)	2.3 (1.72-3.2)	0.001
HCO_3 _(mEq/L)	23.9 (22.8-25.2)	25.6 (21.1-29)	0.159

**Figure 2:**
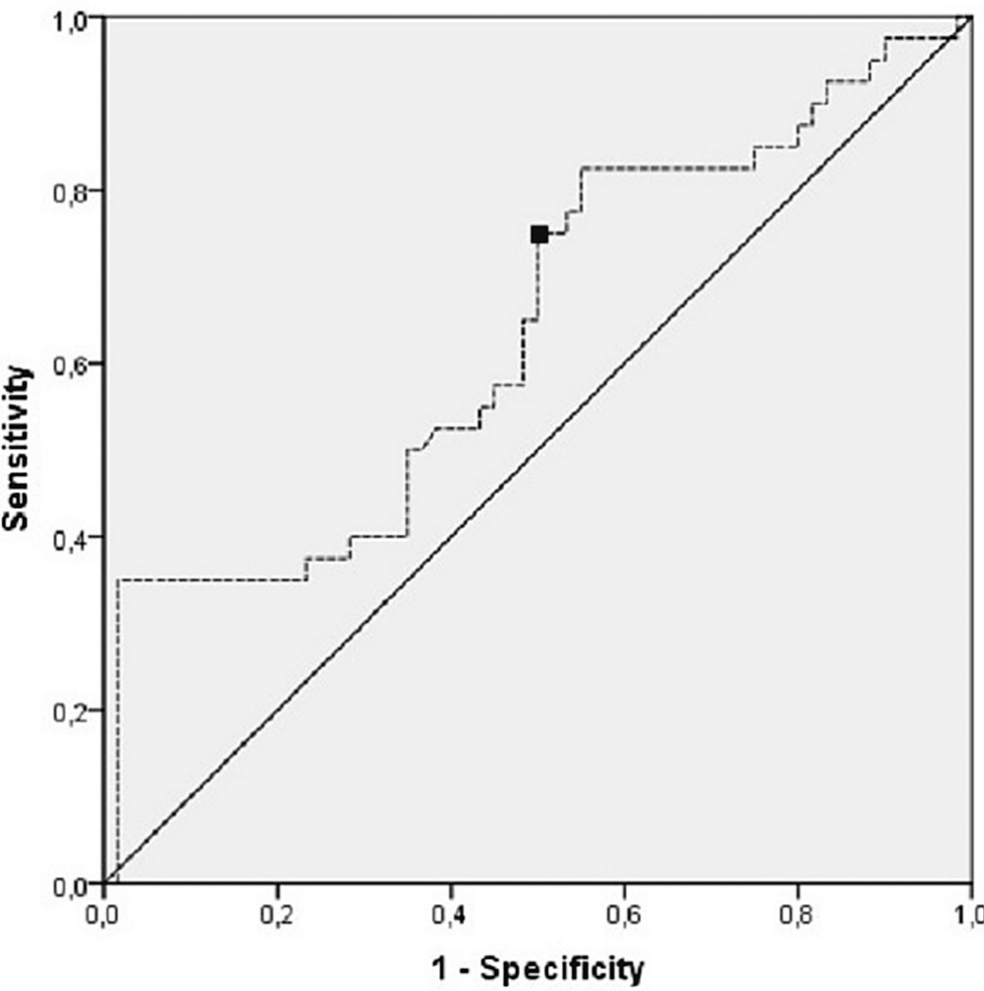
ROC curve analysis of the clinical diagnosis of the patients AUC = 0.622; 95% CI = (0.529-0.714); Cut-off point = 13.23 ng/mL

Using ROC analysis, a larger area under the curve (AUC) for the serum Galectin-3 level of the control group (AUC=0.622, 95% CI = 0.529-0.714; p=0.015) was calculated compared to the COVID-19 patient group for the diagnosis of COVID-19 disease. The Galectin-3 level was found to be 56.9% sensitive and 55.5% specific at a cut-off level of 13.23 ng/mL for the diagnosis of COVID-19 disease (Figure 2).

**Figure 3:**
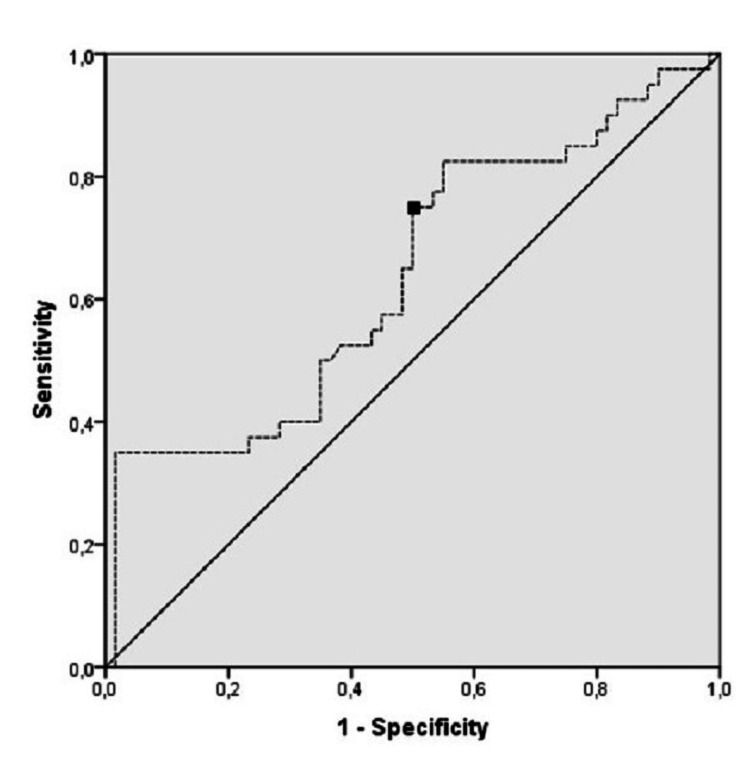
ROC curve analysis of the clinical severity of the patients AUC = 0.639; 95% CI = (0.525-0.752); cut-off point = 11.3 ng/mL

Furthermore, a larger AUC for the serum Galectin-3 levels of patients who have moderate disease (AUC = 0.701, 95% CI = 0.582-0.819; p=0.003) was calculated using ROC analysis. The Galectin-3 level was found to be 75% sensitive and 50% specific at a cut-off level of 11.3 ng/ml for predicting the severity of COVID-19 disease (Figure 3).

## Discussion

This study examined the clinical value of serum Galectin-3 levels in COVID-19 pneumonia and found that serum Galectin-3 levels decreased especially in severe-critical disease patients. The Galectin-3 level was found to be 75% sensitive and 50% specific at a cut-off level of 11.3 ng/mL for predicting the severity of COVID-19 disease.

The Galectin-3 protein, which is known to have an effect on immune system cells and play a role in inflammation, fibrosis, apoptosis and host defense, has been studied in various viral infections in the literature [17]. For example, in their study, Alcendor et al. found that the Galectin-3 level decreased in Kaposi sarcoma-associated herpes virus infection [18]. In another study conducted by King et al., Galectin-3 levels were found to increase HSV infection [19]. In another study, Galectin-3 levels were found to increase chronic hepatitis [20]. All viruses do not have the same pathogenicity. We found different results when we compared this study but it may cause viral entry differences, viral proteins, etc.

There are very few studies examining Galectin-3 levels in pneumonia cases. Chen et al.’s study revealed that Galectin-3 triggered avian H5N1 influenza-induced pulmonary inflammation by activating the NLRP3 inflammasome. They also suggested that Galectin-3 could be a therapeutic target [21].

In Baykan et al.’s study, patients with a diagnosis of COVID-19 infection and typical and atypical pneumonia findings on thorax CT were evaluated and the usability of Galectin-3 level as a diagnostic tool in COVID-19 pneumonia was calculated. The results showed that Galectin-3 level was higher in patients with typical pneumonia findings on thorax CT [14]. In addition, the Galectin-3 level was found to be higher in the patient group than in the control group. However, our study found the Galectin-3 level to be higher in the control group than in the patient group, and the Galectin-3 level was found to be lower, especially in severe-critical patients. We employed a different study design compared to Baykan et al.’s study in that the patients were evaluated by grouping them according to clinical seriousness. For this reason, we think that our study can better reveal the relationship between clinical severity and Galectin-3 level. The lower level in severe-critical patients may be due to a reason such as the release of cytokines during the inflammation process and the consequent consumption of Galectin-3 protein. In this regard, future research can look at the sequential Galectin-3 levels during the clinical follow-up of the patients. In addition, our study sheds light on the clinical status of the Galectin-3 protein level, which was suggested to be a treatment target in Chen et al.’s study.

Regarding the use of Galectin-3 level as a prognostic and diagnostic marker, Baykan et al. found Galectin-3 level to be important in diagnosing typical pneumonia and the AUC value to be 0.89 (95% CI = 0.83-94), and no association was made with clinical seriousness of the patients. However, in our study, we found lower AUC values ​​in diagnosing COVID-19 disease and predicting severity. Although AUC=0.622 (95% CI =0.529-0.714; p=0.015) for the control group and AUC = 0.701 (95% CI = 0.582-0.819; p=0.003) for the moderate disease group were not higher than those in our study, that Galectin-3 level was found to be 75% sensitive and 50% specific at a value of 11.3 ng/mL indicates that it can be a diagnostic marker in the prognosis of the disease.

This study has some limitations. Firstly, we did not measure Galectin-3 levels continuously, and we have no data about the continuous changing of the Galectin-3 levels. Secondly, we did not follow up with the patients about mortality.

## Conclusions

Many markers have been studied both in the diagnosis of COVID-19 and in predicting prognosis, and Galectin-3 can be used more appropriately for clinical classification of patients rather than diagnosis. Especially low Galectin-3 levels detected in severe-critical disease groups may shed light on the timing of Galectin-3 protein-targeted therapies. Galectin-3 levels may be a beneficial biomarker in predicting the clinical severity of COVID-19 disease when used in conjunction with other known biomarkers, at the time of admission to the ED.
